# Memory maintenance in calcium-based plastic synapses in the presence of background activity

**DOI:** 10.1186/1471-2202-14-S1-P42

**Published:** 2013-07-08

**Authors:** David C Higgins, Michael Graupner, Nicolas Brunel

**Affiliations:** 1 IBENS, École Normale Supérieure, Paris, France; 2 Departments of Statistics and Neurobiology, University of Chicago, USA; 3 Center for Neural Science, New York University, New York, USA

## 

How are synaptic modifications, elicited by specific patterns of activity, stable in the face of ongoing background activity? We investigate this question using a calcium-based synaptic plasticity model that is able to fit a wide range of data on various spike-timing dependent plasticity (STDP) protocols, in various in-vitro preparations (Graupner and Brunel, 2012). One of the advantages of this model is that it is possible to compute analytically how average synaptic strengths evolve in time, in the presence of random pre- and post-synaptic Poisson firing.

We analyse the synapse behaviour using two parameter sets: an 'in-vitro' set, with parameters that best fit cortical slice STDP experiments (Sjostrom et al, 2001), obtained with an extracellular calcium concentration of 2.5 mM; an 'in-vivo' set, adjusting parameters of the model to in-vivo level extracellular calcium concentrations (1.5 mM, Silver and Erecinska, 1990).

We start by analysing a model with no bistability at resting intracellular calcium concentrations. We find that the memory of the initial state decays exponentially, with a time constant that is inversely proportional to the background firing rate, according to a power law with an integer exponent that depends on the relative sizes of the calcium transients triggered by pre- and post-synaptic spikes with respect to the depression threshold. Importantly, the predicted memory timescales for the 'in-vivo' parameter set is several orders of magnitude longer than that for the 'in-vitro' set (hours vs minutes, for a 1/s background rate).

We extend the analysis to examine the effect of synaptic bistability for memory retention. We find that under low frequency background firing (<1 Hz), bistable synapses in-vivo retain their memories for long periods (~months).

Results are found to be qualitatively similar in simulations of an isolated pair of Poisson neurons, and in large-scale simulations of a network of excitatory and inhibitory neurons, operating at low rates. Our results demonstrate the role of synaptic bistability for memory retention in cortical circuits in the presence of realistic background activity.

